# In Vivo Foot Segmental Motion and Coupling Analysis during Midterm Follow-Up after the Open Reduction Internal Fixation of Trimalleolar Fractures

**DOI:** 10.3390/jcm12082772

**Published:** 2023-04-07

**Authors:** Harm Hoekstra, Olivier Vinckier, Filip Staes, Lisa Berckmans, Jolien Coninx, Giovanni Matricali, Sander Wuite, Eline Vanstraelen, Kevin Deschamps

**Affiliations:** 1 Department of Trauma Surgery, University Hospitals Leuven, Herestraat 49, 3000 Leuven, Belgium; 2 Department of Development and Regeneration, KU Leuven—University of Leuven, 3000 Leuven, Belgium; 3 Department of Orthopaedics, University Hospitals Leuven, Herestraat 49, 3000 Leuven, Belgium; 4 Musculoskeletal Rehabilitation Research Group, Department of Rehabilitation Sciences, KU Leuven—University of Leuven, Tervuursevest 101, 3001 Leuven, Belgium; 5 Institute for Orthopaedic Research and Training, KU Leuven—University of Leuven, Herestraat 49, 3000 Leuven, Belgium; 6 Clinical Motion Analysis Laboratory, Campus Pellenberg, University Hospitals Leuven, Weligerveld 1, 3212 Lubbeek, Belgium; 7 Division of Podiatry, Institut D’Enseignement Supérieur Parnasse Deux-Alice, Haute Ecole Leonard de Vinci, Avenue e Mounier 84, 1200 Bruxelles, Belgium; 8 Department of Podiatry, Artevelde University College, Hoogpoort 15, 9000 Gent, Belgium

**Keywords:** coupling analysis, foot segmental motion, trimalleolar ankle fractures

## Abstract

Purpose: Trimalleolar ankle fractures (TAFs) are common traumatic injuries. Studies have described postoperative clinical outcomes in relation to fracture morphology, but less is known about foot biomechanics, especially in patients treated for TAFs. The aim of this study was to analyze segmental foot mobility and joint coupling during the gait of patients after TAF treatment. Methods: Fifteen patients, surgically treated for TAFs, were recruited. The affected side was compared to their non-affected side, as well as to a healthy control subject. The Rizzoli foot model was used to quantify inter-segment joint angles and joint coupling. The stance phase was observed and divided into sub-phases. Patient-reported outcome measures were evaluated. Results: Patients treated for TAFs showed a reduced range of motion in the affected ankle during the loading response (3.8 ± 0.9) and pre-swing phase (12.7 ± 3.5) as compared to their non-affected sides (4.7 ± 1.1 and 16.1 ± 3.1) and the control subject. The dorsiflexion of the first metatarsophalangeal joint during the pre-swing phase was reduced (19.0 ± 6.5) when compared to the non-affected side (23.3 ± 8.7). The affected side’s Chopart joint showed an increased range of motion during the mid-stance (1.3 ± 0.5 vs. 1.1 ± 0.6). Smaller joint coupling was observed on both the patient-affected and non-affected sides compared to the controls. Conclusion: This study indicates that the Chopart joint compensates for changes in the ankle segment after TAF osteosynthesis. Furthermore, reduced joint-coupling was observed. However, the minimal case numbers and study power limited the effect size of this study. Nevertheless, these new insights could help to elucidate foot biomechanics in these patients, adjusting rehabilitation programs, thereby lowering the risk of postoperative long-term complications.

## 1. Introduction

Ankle fractures are relatively common musculoskeletal injuries with an average incidence of 168.7/100,000/year [1]. The posterior tibia is involved in almost half of the Weber type B or C ankle fracture dislocations [2]. In most cases, a high-energy trauma is the primary cause of a trimalleolar ankle fracture (TAF), wherein both the medial, lateral, and posterior malleolus are involved. The trauma-mechanism-based Lauge–Hansen classification has been used for many years in guiding treatment and predicting instability. This system is built on a comprehensive understanding of trauma mechanism and the interplay between fracture morphology and ligamentous injury. The open reduction and internal fixation of the posterior malleolus, via either a posterolateral or posteromedial approach, is most commonly applied in our center for these fractures [3]. However, there is still a lack of consensus on whether the fixation of the posterior malleolus is always necessary. Treatment strategies that take the size and displacement of the posterior fracture fragment into consideration remain an issue of debate [4,5,6].

TAFs and subsequent plate osteosynthesis are associated with osteoarthritis, arthro-fibrosis, and fibro-adhesions (i.e., flexor hallucis longus and peroneal muscles), leading to restriction in ankle joint mobility [7,8,9,10]. Subsequently, alterations in foot joint mobility, segment coupling (kinematic relationship between adjacent foot segments), and kinetics (forces acting on the foot joints) during gait may occur as well. It has been theorized that altered or disrupted coupling mechanisms may contribute to poor functional outcome scores due to pathological joint contact forces and soft tissue stress [11,12]. Nevertheless, there is a lack of in vivo dynamic assessments on the foot which specific focus on the functional outcomes of patients with a history of TAF osteosynthesis. Previous studies that reported on foot kinematics after the operative treatment of ankle fractures consisted of heterogeneous groups and used the Oxford foot model, which does not include the midfoot as a separate segment [13,14].

Therefore, we aimed to measure the segmental foot mobility and joint coupling of patients with a history of a TAF osteosynthesis. The affected side was compared to the non-affected contralateral side, as well as with a healthy control group. We hypothesized a reduction in the affected sides’ hindfoot range of motion (ROM) during the loading response and the pre-swing of the stance phase, with most distinct changes seen in the frontal plane and sagittal plane, respectively. Furthermore, we hypothesized a reduced joint coupling between hindfoot and shank, as well as between the hindfoot and the forefoot.

## 2. Materials and Methods

Level of Evidence: III.

### 2.1. Patients

Fifteen patients who sustained a TAF (AO/OTA type B3) and underwent open reduction and internal fixation were retrospectively recruited. All patients underwent surgery between 2015 and 2018 at the Trauma Surgery department of the University Hospitals Leuven. Five patients had a luxation of the tibiotalar joint and two patients had a subluxation of the tibiotalar joint. None of the patient group had an open fracture. Polytrauma was not included in our study population. In total, 10 of the 15 patients obtained a temporary external fixator awaiting definitive internal fixation. The posterior malleolus and distal fibula fracture were addressed using plate screw osteosynthesis via a posterolateral approach, whereas the medial malleolus fracture was fixated using screws via a medial approach. Standardly one-third tubular plates with the small fragment system screws by DePuy Synthes™ (Raynham, MA, USA) were used to achieve anatomical reduction and internal fixation. If deemed necessary by the treating surgeon, variable angle LCP^®^ was utilized in some cases. Additional syndesmotic screw fixation was performed if syndesmotic instability persisted after osseous fixation. Postoperatively, a fixed protocol was followed, consisting of immediate passive and active mobilization and toe-touch weight bearing (<10 kg) for 6 weeks. The control group consisted of 13 healthy subjects that were chosen at random from an existing database set-up in the same laboratory [6]. We matched a TAF subject to a control patient of the same gender, similar age, and walking speed to avoid the influence of confounding factors. An a priori sample size calculation showed that at least 12 patients and 12 control subjects were required to assure a minimal study power of 80% (β > 0.80). This calculation was based on biomechanical parameters reported in other studies, including patients with ankle osteoarthritis or ankle fractures [13,15]. Informed consent was obtained from all participants and the ethical committee of the University Hospitals Leuven (S62064) approved the study.

### 2.2. 3D Gait Analysis

In this study, a 3D gait analysis was performed in the Clinical Motion Analysis Laboratory of the University Hospitals Leuven Belgium. The analysis was performed using a multi-segment foot model where reflecting skin-markers were placed according to the Rizzoli foot model marker placement protocol using double-sided tape [16]. This multi-segment foot model calculates the 3D rotation between adjacent segments of the foot and tibia. For reasons of readability, these inter-segment angles will be reported with respect to their corresponding anatomical joints, i.e., the ankle segment (consisting of two joint levels: tibiotalar and subtalar), the Chopart joint, the Lisfranc joint, and the first metatarsophalangeal (MTP 1) joint. Kinematic data were captured with a passive optoelectronic measurement, including ten infrared cameras (T10, 100 Hz, Vicon Motion Systems Ltd., Oxford Metrics, UK). For this study, only the stance phase of gait was considered, which was delineated with initial contact as the start and toe-off as the end. The measurements were taken on the symptomatic side as well as the asymptomatic side. The first measurement was taken as a static recording and was used as a reference position. After this, the dynamic measurements were recorded. The participants were asked to repeatedly walk along a 10 m walkway at a self-selected speed until five representative trials were registered. The walkway was instrumented with a force plate (Advanced Mechanical Technology Inc., 200 Hz, Watertown, MA, USA) in order to determine the gait events such as the initial contact and the toe-off. A plantar pressure plate (Footscan™, dimensions 0.5 m × 0.4 m, 4096 sensors, 2.8 sensors per cm^2^, RSscan International, Olen, Belgium) was placed on top of the force plate. Plantar pressure and force data were synchronized with a 3D box™ (RSscan International, Olen, Belgium) using an external trigger. These data were sampled at 200 Hz.

### 2.3. Data Processing and Analysis

The 3D inter-segment joint angles and joint coupling were calculated for the stance phase (0–62%) of the gait cycle. The swing phase of gait (63–100%) was not considered. In-house-made software (ACEPManager, Matlab 2016a, The Mathworks Inc., Natick, MA, USA) was used to normalize the time of the kinematic data to a 100% stance phase during the gait events. Kinematic variables of interest were subsequently calculated. We distinguished 4 sub-phases of the stance phase, i.e., the “loading response” (0–12%), “mid stance” (13–30%), “terminal stance” (31–50%), and “pre-swing” (51–62%) phases. Kinematic variables of interest were subsequently determined by calculating the ROM in each sub-phase. The latter was carried out by calculating the difference between the maximum and minimum value in the respective sub-phase. Furthermore, the patient-reported outcome measures, using the ankle–hindfoot scale (AOFAS), the EuroQol health scale (EQ-5D), and the EQ-5D visual analogue scale (VAS), were evaluated. The AOFAS monitors the progression of patients after foot and ankle surgery, the EQ-5D records the health state as rated by the caregiver, and the EQ-5D VAS assumes the individual’s health state valuation [17,18].

### 2.4. Statistical Analysis

Statistical analysis was performed with SPSS Statistics 27.0 (IBM Corp., New York, NY, USA). When comparing the affected side to the non-affected side of the same subject, a test of normality, the Shapiro–Wilk test (α = 0.05), was completed. For the variables that were not normally distributed, a Wilcoxon signed-rank test (α = 0.05) was used to compare the samples. The other variables were statistically compared using a paired *t*-test t (α = 0.05). A univariate analysis of variance (ANOVA) was used to compare the control group with the affected group. The same analysis was performed with the inclusion of the BMI as a covariate, since the BMIs of the patients were found to be significantly higher than those in the control group.

To guard against the inflation of a type I error but maintain statistical power across the multiple comparisons, an adjusted alpha level was applied by dividing the alpha level by the number of parameters (*p* = 0.05/4 = 0.0125). Additionally, the effect size was determined for each difference (Cohen’s d for the *t*-tests, r for the Wilcoxon tests, and partial η^2^ for the ANOVA tests). A large effect size can be interpreted when d ≥ 0.8, r ≥ 0.5, and η^2^ ≥ 0.25 [19,20].

To evaluate the level of joint coupling between a number of inter-segment angles, cross-correlation coefficients were calculated based on the 1D waveforms associated with the normalized stance phases [21]. The following four joint couplings were analyzed: ankle inversion–eversion with ankle adduction–abduction, ankle inversion–eversion with forefoot dorsal flexion–plantar flexion, ankle inversion–eversion with forefoot inversion–eversion, and ankle inversion–eversion with forefoot adduction–abduction. This selection was based on a previous publication, which showed the high correlation between these four inter-segment rotations [22]. When assessing the cross-correlation, the following benchmarks were used: a large joint coupling >0.7 or <−0.7, a medium joint coupling between (−) 0.3 and (−) 0.69, and a small joint coupling between −0.3 and 0.3 [22].

## 3. Results

### 3.1. Demographics

Demographic characteristics of the patient group and control group are presented in Table 1. The two groups had a similar age, length, and gender ratio. The walking speed did not differ significantly between the two groups. The TAF group had a significantly higher body mass index (BMI) compared to the control group (*p* < 0.001). The BMI was therefore considered as a confounding factor when analyzing the kinematic differences between these two groups. Gait analysis was performed for 29 months after surgery on average. Five patients were treated with a syndesmotic screw, because insufficient syndesmotic stability of the distal tibiofibular joint was achieved with the plate osteosynthesis of the posterior malleolus alone. The mean clinical follow-up lasted 101 weeks (range: 60–171). Seven of the fifteen patients underwent some kind of implant removal. One patient had a superficial wound infection after implant removal. One patient developed a chronic regional pain syndrome (CRPS). No non-unions or mal-unions were reported. Two patients showed progression towards tibiotalar osteoarthritis on the latest available X-rays.

### 3.2. Patient-Reported Outcome Scores

The AOFAS, EQ-5D, and EQ-5D VAS scores are displayed in Table 2 with missing data for three patients [7]. The patients scored an average of 78 on the AOFAS survey, reflecting a good outcome after surgery (e.g., 100 = excellent score). For the first domain of the EQ-5D, mobility, the majority (67%) of the patient group (TAF) had moderate problems. None of the 12 patients reported self-care problems. The minority of patients (42%) suffered problems with daily activities, whereas two-thirds of the patients experienced moderate pain (67%). The presence of depression/anxiety was found to be negligible.

### 3.3. ROM Comparisons of the Affected and Contralateral Side in the Patient Group

**Figure 1:**
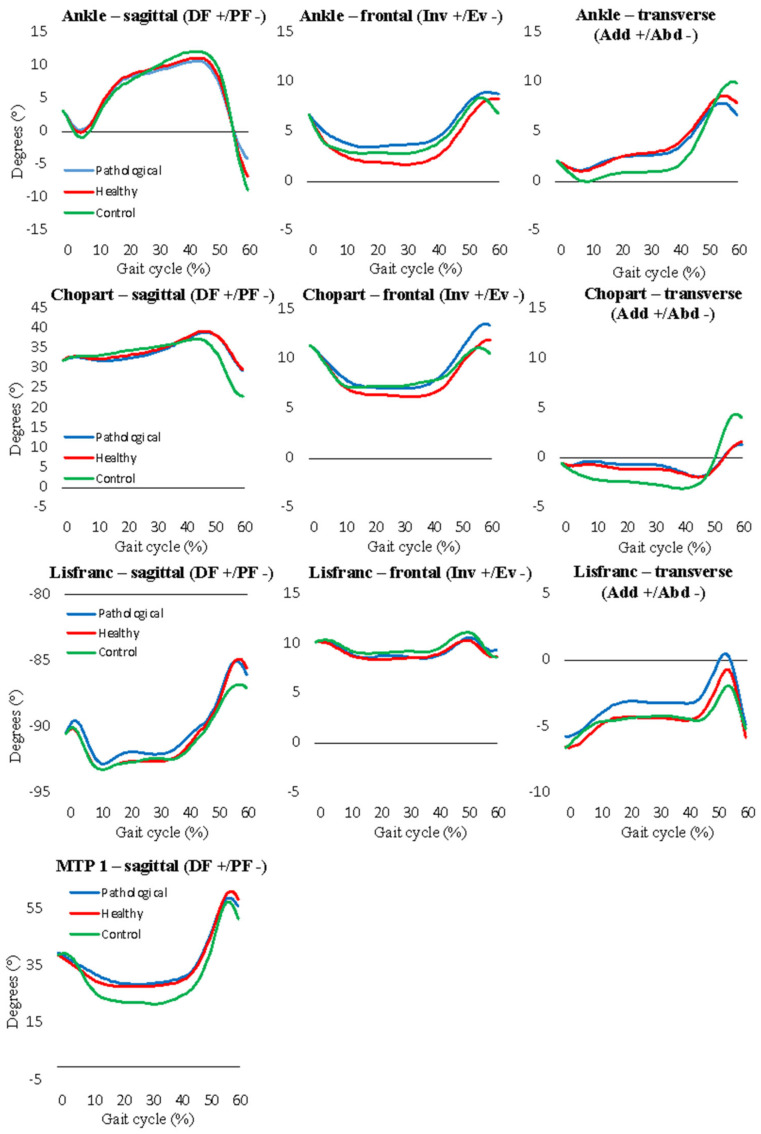
Kinematic waveforms of the ankle, Chopart, Lisfranc, and MTP 1 joints. Abbreviations: DF, dorsal flexion; PF, plantar flexion; Inv, inversion; Ev, eversion; Add, adduction; Abd, abduction.

During the loading response, the ankle segment of the affected side presented a significantly reduced ROM in the sagittal plane (*p* < 0.0125, large effect) and a trend towards reduced frontal plane ROM (*p* = 0.049, medium effect) (Table 3, Figure 1). Furthermore, the Chopart joint of the affected foot showed a trend towards reduced frontal plane ROM during the loading response (*p* = 0.013) and a trend towards an increased ROM during the midstance phase (*p* = 0.03). No significant differences were observed during the terminal stance phase. During the pre-swing phase, the mean ankle segment sagittal and transverse plane ROM was significantly reduced (*p* < 0.0125) in the affected foot, with mean differences of −3.4° and −1.4° and effect sizes of d = −0.9 (large) and d = −0.5 (medium), respectively.

### 3.4. Comparison ROM Affected Side in the Patient Group versus Control Group

During the loading response, the transverse plane ankle segment ROM was significantly (*p* < 0.0125) lower in the affected side (1.3 ± 0.6) compared to the control group (3.1 ± 1.1), with a large effect (η^2^ = 0.37) (Table 4, Figure 1). During the midstance phase, the affected foot showed a reduced transverse plane ROM at the Chopart joint; hence, this did not reach a significance level. During the terminal stance phase, the affected foot demonstrated a reduced (*p* = 0.045) sagittal plane ROM at the ankle segment and an increased ROM in the Chopart joint (*p* = 0.038).

During the pre-swing phase, the affected foot demonstrated a significantly reduced transverse plane ROM compared to the control group (*p* = 0.011, medium effect). Moreover, a clear trend towards reduced ROM was observed in other joints (ankle segment and Chopart joint) and planes (sagittal and transverse) as well.

### 3.5. Comparison Joint Coupling for the Three Cohorts

The cross-correlation coefficients showed a small joint coupling for the patient group in both feet when compared to the control group (Table 5). The largest differences were seen for the following inter-segment rotations: ankle inversion–eversion with ankle adduction–abduction, ankle inversion–eversion with forefoot dorsiflexion–plantarflexion, and ankle inversion–eversion with forefoot inversion–eversion. For these inter-segment rotations, the patient group showed a medium joint coupling ((−) 0.3 to (−) 0.69) for the non-affected and affected side, while the control group showed large joint coupling (>0.7 or <−0.7).

## 4. Discussion

In this study, the segmental ROM and coupling of the foot joints were compared between patients that were operatively treated for a TAF and a control group. Only two previous studies have reported on foot kinematics after the operative treatment of ankle fractures. However, the patient cohorts were heterogeneous and the used Oxford foot model did not include the midfoot as a separate segment [13,14]. Moreover, both studies investigated coupling among the different segments of the foot.

In our study, we observed a general trend towards reduced ROM and joint coupling of the affected foot, particularly the ankle segment and midfoot joints. The patient group presented a mean body mass index corresponding to obesity class I, which is an interesting finding. From a functional viewpoint, it is reasonable to assume that an elevated BMI can be considered as a risk factor for moderate and low impact trauma, as frequently seen in TAFs. Nevertheless, further large-scale population studies are necessary to validate this assumption.

General information provided by the AOFAS score leads to the conclusion that the patients included in the population faced moderate pain and mobility problems at the onset of the investigation. The biomechanical outcome measures quantified here could thus also be (partly) explained by these patient’ reported outcome measurements.

When comparing the patients’ affected side to the contralateral non-affected side, some differences were obvious. The changes observed in the ankle segment during the loading response and the pre-swing phase can be a result of possible arthritis and arthro-fibrosis of the ankle joint and fibro-adhesions (i.e., muscle adhesions) due to surgery, which all affect ankle joint mobility [7,8,9,10]. Additionally, the involvement of pain at the affected foot may also contribute to the reduced ROM seen during the loading response and the pre-swing phase. The reduced ROM during the pre-swing phase may originate from the weakness of the calf muscles on the one hand, but could also be associated with a (mal)adaptive strategy of the patient in order to avoid peak loading in the posterior part of the ankle joint.

The Chopart joint of the affected foot had a significantly reduced ROM in the frontal plane during the loading response. A similar observation has been reported by Eerdekens et al. in patients with ankle osteoarthritis. This tibiotalar stiffness leads to the conclusion that this reduced motion is possibly associated with co-contraction and a more cautious walking strategy [23].

During the pre-swing phase, the MTP 1 joint of the affected foot showed a reduced ROM (mainly dorsal flexion) (Table 3 and Figure 1). This restriction may consequently be caused by muscle adhesions (fibro-adhesions) of the flexor hallucis longus muscle due to posterior plate osteosynthesis. However, from a functional viewpoint, this finding may also highlight a suboptimal usage of the “windlass mechanism” during propulsion, which in turn may affect the physiological joint coupling among the joints of the foot [24].

When comparing the affected foot of the patient group to the control group, a significant reduced transverse plane ankle ROM was quantified during the loading response. The latter observation explains the medium joint coupling observed between ankle inversion–eversion and adduction–abduction. Potential causes for this medium joint coupling may be due to arthro-fibrosis and fibro-adhesions at the posterior aspect of the ankle, the presence of co-contraction of extrinsic foot muscles, or alterations in foot placement during initial contact.

Differences in the ROM were also observed in the ankle segment and Chopart joint during the midstance, terminal stance, and pre-swing phases. Therefore, it can be concluded that there is less plantar flexion during propulsion in these joints (Figure 1) and the hindfoot tends to maintain a more abducted position. A similar observation was reported in the previous study of van Hoeve et al. [13]. Such a reduced plantar flexion was also observed when comparing the affected foot with the unaffected foot, which seems to point towards the presence of weakness of the calf muscles on the one hand or the presence of a (mal)adaptive strategy, avoiding any peak loading in the posterior aspect of the talus and posterior malleolus.

In the current study, small joint coupling was observed in the affected foot. One may hypothesize that this could be caused by perturbed neuromuscular control, proprioception, or the presence of arthro-fibrosis and muscle adhesions at the posterior aspect of the ankle. Despite the fact that these assumptions are realistic and logic, it should be recognized that a similar level of joint coupling was observed in the non-affected side of the patient group. This was an unexpected finding in the study and raises two new hypotheses. The first concerns whether the unaffected limb adopts a (mal)adaptive movement pattern to strive for gait symmetry. This adaptation is also seen in the knee after ACL reconstruction, where kinematic differences between the ACL-reconstructed limb and contralateral unaffected limb decrease over time because of alterations in both limbs [25]. A second hypothesis proposes that this smaller joint coupling was a pre-existing biomechanical phenomenon prior to the ankle trauma and that, together with the increased body mass, it can be considered as a risk factor for the development of ankle fractures. Further research is needed validate to validate or reject this hypothesis.

The postoperative rehabilitation for patients treated for a TAF needs to place emphasis on regaining full ankle joint mobility with passive and active exercises, as well as early protective weight bearing. Full plantar flexion mobility should be highlighted and transferred into the gait pattern, with a focus on the pre-swing phase. Gait training is thereby an important aspect of rehabilitation. Previous studies have concluded that early postoperative mobilization and weight bearing is safe and does not increase the complication rate in patients treated for ankle fractures [26,27,28]. Combining these aspects with weight reduction could lower the risk of developing post-traumatic osteoarthritis. Patients need to receive a home exercise program so that daily practice is possible and the patient can be autonomous in their treatment.

An important limitation of the current study is the non-standardized period between surgery and gait analysis. Within the patient group, the range was between 8 and 49 months after the operation. The differences in time between both events will influence the collected data because of dissimilarities in the recovery time. In addition, the low case numbers and the minimal study power result in limited practical applications. Another limitation is the condition of barefoot walking when the data were collected. When wearing shoes, the kinematic data may differ from the data during barefoot walking [29]. Lastly, this study only observed patients during walking. Therefore, these outcomes are not representable for more challenging tasks, e.g., running or jumping. It is hypothesized that more complex and challenging tasks may unravel other biomechanical differences than those reported here.

## 5. Conclusions

This study found that patients with a history of a TAF show reduced ROM in the affected ankle segment during the loading response and the pre-swing phase compared to their non-affected side and control subject. The affected sides’ Chopart joint showed increased ROM during midstance to compensate for reduced ankle segment ROM during loading responses. Finally, small joint coupling was observed in the affected side as well as the non-affected side compared to the control group. Despite the limited effect size of our results, the findings of this study emphasize the importance of adequate postoperative rehabilitation to restore mobility and thereby potentially lower the risk of post-traumatic osteoarthritis in patients with a history of TAFs.

## Figures and Tables

**Table 1:** Baseline characteristics of participants.

	Patient Group (*n* = 15)	Control Group (*n* = 13)	*p*
Age (years)	54 (37–68)	47 (33–64)	0.037
Length (cm)	168.5 (151.0–190.3)	171.2 (158.0–193.3)	
Body mass (kg)	86.2 (58.6–120.3)	67.9 (54.0–95.0)	0.003
BMI (kg/m^2^)	30.1 (22.9–37.7)	23.1 (19.5–26.6)	<0.001
Gender (male/female)	6/9	6/9	
Walking speed (m/s)	1.2 (1.0–1.4)	1.2 (1.0–1.4)	0.914
Months until post-surgical gait analysis	29 (8–49)		
Operative characteristics			
Side (L/R)	8/7		
Posterior plate fixation	15 (100%)		
Medial screw fixation	15 (100%)		
Fibula plate fixation	15 (100%)		
Syndesmotic screw	5 (33.3%)		

Continuous variables are expressed as means and ranges and categorical variables are presented as numbers and percentages. Abbreviations: L, left; R, right.

**Table 2:** Patient-reported outcomes after trimalleolar ankle fracture osteosynthesis.

	Patient Group (*n* = 12)
AOFAS score	78.3 (59.0–100)
EQ-5D score	
*Mobility*	
No problems	4 (33.3%)
Moderate problems	8 (66.7%)
Extreme problems	/
*Self-care*	
No problems	12 (100%)
Moderate problems	/
Extreme problems	/
*Activities*	
No problems	7 (58.3%)
Moderate problems	5 (41.7%)
Extreme problems	/
*Pain/discomfort*	
No problems	4 (33.3%)
Moderate problems	8 (66.7%)
Extreme problems	/
*Depression/anxiety*	
No problems	11 (91.7%)
Moderate problems	1 (8.3%)
Extreme problems	/
EQ-5D VAS	81.0 (50.0–100)

Categorical variables are expressed as numbers and percentages. The AOFAS is expressed as the mean and range. All questionnaires were standardized and tested for reproducibility and validated for the Dutch language. Three of the fifteen patients did not report their outcome scores. Abbreviations: AOFAS, American Orthopedic Foot and Ankle Society; EQ-5D, EuroQol 5 dimensions; VAS, visual analog scale.

**Table 3:** Patient group ROM of the affected foot and non-affected contralateral sides.

			Affected Foot	Non-Affected Foot	Mean Difference		
			*n* = 15	*n* = 15	[95% CI]	*p*	Effect Size
Loading response	Ankle	DF/PF	3.8 ± 0.9	4.7 ± 1.1	−0.9 [−1.6; −0.2]	0.010	−0.8
		Inv/Eve	2.9 ± 1.5	4.3 ± 1.9	−1.3 [−2.6; 0.0]	0.049	−0.6
		Add/Abd	1.3 ± 0.6	2.0 ± 1.2	−0.7 [−1.4; 0.1]	0.079	−0.5
	Chopart	DF/PF	1.8 ± 1.2	2.0 ± 1.3	−0.2 [−0.7; 0.3]	0.410	−0.2
		Inv/Eve	3.4 ± 0.9	4.3 ± 1.3	−0.8 [−1.5; −0.2]	0.013	−0.7
		Add/Abd	1.3 ± 0.9	1.2 ± 0.8	0.1 [−0.6; 0.8]	0.594	−0.1
	Lisfranc	PF/DF	3.6 ± 1.3	3.4 ± 1.5	0.2 [−0.6; 1.0]	0.551	−0.1
		Inv/Eve	1.8 ± 1.1	2.0 ± 1.1	−0.1 [−0.7; 0.5]	0.617	−0.1
		Add/Abd	2.5 ± 0.9	2.5 ± 1.1	0.0 [−0.7; 0.7]	0.970	−0.0
	MTP 1	DF/PF	8.0 ± 3.0	8.8 ± 7.0	−0.8 [−3.6; 1.9]	0.533	−0.2
Mid-stance	Ankle	DF/PF	5.2 ± 1.4	5.5 ± 1.6	−0.3 [−1.0; 0.3]	0.296	−0.3
		Inv/Eve	1.2 ± 0.6	1.6 ± 1.1	−0.4 [−1.1; 0.3]	0.683	−0.1
		Add/Abd	1.7 ± 1.3	2.3 ± 1.5	−0.6 [−1.4; 0.2]	0.109	−0.3
	Chopart	DF/PF	1.9 ± 0.7	2.0 ± 0.7	−0.1 [−0.5; 0.4]	0.707	−0.1
		Inv/Eve	1.3 ± 0.5	1.1 ± 0.6	0.3 [0.0; 0.5]	0.030	0.6
		Add/Abd	0.7 ± 0.5	0.7 ± 0.4	0.0 [−0.6; 0.6]	0.638	−0.1
	Lisfranc	DF/PF	1.2 ± 0.7	1.0 ± 0.6	0.2 [−0.4; 0.8]	0.510	0.1
		Inv/Eve	0.7 ± 0.5	0.7± 0.3	0.0 [−0.6; 0.6]	0.510	−0.1
		Add/Abd	1.1 ± 0.8	0.8 ± 0.6	0.3 [−0.4; 1.0]	0.140	−0.3
	MTP 1	DF/PF	4.0 ± 3.5	3.2 ± 2.5	0.8 [−0.1; 1.7]	0.778	−0.1
Terminal stance	Ankle	DF/PF	2.6 ± 1.2	2.5 ± 1.4	0.1 [−0.4; 0.7]	0.587	0.1
		Inv/Eve	3.6 ± 1.3	3.7 ± 1.4	−0.9 [−0.8; 0.7]	0.815	−0.1
		Add/Abd	3.7 ± 1.8	3.9 ± 1.8	−0.2 [−1.0; 0.6]	0.580	−0.1
	Chopart	DF/PF	5.5 ± 1.5	5.3 ± 1.6	0.2 [−0.6; 1.0]	0.582	0.1
		Inv/Eve	3.2 ± 1.2	2.8 ± 1.1	0.5 [−0.1; 1.0]	0.105	0.4
		Add/Abd	1.4 ± 0.7	1.3 ± 0.6	0.1 [−0.4; 0.7]	0.592	0.1
	Lisfranc	DF/PF	3.2 ± 1.3	3.5 ± 1.1	−0.3 [−1.0; 0.4]	0.177	−0.2
		Inv/Eve	1.9 ± 0.9	1.9 ± 1.0	0.0 [−0.6; 0.6]	0.992	0.0
		Add/Abd	2.6 ± 1.9	2.4 ± 1.4	0.3 [−0.5; 1.1]	0.465	0.2
	MTP 1	DF/PF	11.7 ± 4.8	11.7 ± 5.7	−0.1 [−2.3; 2.2]	0.960	−0.0
Pre-swing	Ankle	DF/PF	12.7 ± 3.5	16.1 ± 3.1	−3.4 [−5.6; −1.3]	0.004	−0.9
		Inv/Eve	2.3 ± 1.5	3.0 ± 1.6	−0.7 [−1.5; 0.1]	0.211	−0.2
		Add/Abd	2.3 ± 1.5	3.7 ± 2.9	−1.4 [−2.3; −0.5]	0.011	−0.5
	Chopart	DF/PF	9.5 ± 2.1	9.7 ± 2.6	−0.2 [−1.2; 0.9]	0.750	−0.1
		Inv/Eve	3.4 ± 1.8	3.4 ± 1.7	0.0 [−0.9; 0.8]	0.931	−0.0
		Add/Abd	3.1 ± 1.6	3.6 ± 2.1	−0.4 [−1.5; 0.7]	0.426	−0.2
	Lisfranc	DF/PF	4.5 ± 1.8	4.5 ± 1.8	0.0 [−0.8; 0.8]	0.470	−0.1
		Inv/Eve	2.1 ± 1.4	2.4 ± 1.7	−0.3 [−1.1; 0.5]	0.820	−0.0
		Add/Abd	5.9 ± 2.5	5.8 ± 3.1	0.1 [−1.2; 1.4]	0.836	0.1
	MTP 1	DF/PF	19.0 ± 6.5	23.3 ± 8.7	−4.5 [−8.5; −0.4]	0.040	−0.6

*p* values represent the outcome of the paired *t*-test (α = 0.05); significance: *p* < 0.0125; when data were not normally distributed, the *p* values represent the outcome of the Wilcoxon test. Effect size represents the Cohen’s d calculated for the *t*-test and r-value for the Wilcoxon test. Abbreviations: MTP 1, first metatarsophalangeal joint; DF/PF, dorsal flexion–plantar flexion (sagittal plane); Inv/Eve, inversion–eversion (frontal plane); Add/Abd, adduction–abduction (transverse plane).

**Table 4:** ROM of the affected foot versus the control group.

			Affected Foot	Controls	Mean Difference		
			*n* = 15	*n* = 13	[95% CI]	*p*	Partial η^2^
Loading response	Ankle	DF/PF	3.8 ± 0.9	5.1 ± 1.6	−1.3 [−2.1; −0.5]	0.096	0.11
		Inv/Eve	2.9 ± 1.5	3.9 ± 1.5	−0.9 [−1.7; −0.1]	0.872	0.00
		Add/Abd	1.3 ± 0.6	3.1 ± 1.1	−1.8 [−2.5; −1.1]	0.001	0.37
	Chopart	DF/PF	1.8 ± 1.2	3.1 ± 4.4	−1.3 [−2.2; −0.4]	0.539	0.02
		Inv/Eve	3.4 ± 0.9	4.4 ± 2.3	−1.0 [−1.8; −0.2]	0.172	0.07
		Add/Abd	1.3 ± 0.9	2.6 ± 2.8	−1.3 [−2.2; −0.4]	0.150	0.08
	Lisfranc	DF/PF	3.6 ± 1.3	3.6 ± 1.7	0.0 [−0.8; 0.8]	0.378	0.03
		Inv/Eve	1.8 ± 1.1	2.3 ± 1.2	−0.4 [−1.2; 0.4]	0.586	0.01
		Add/Abd	2.5 ± 0.9	2.3 ± 1.2	0.2 [−0.6; 1.0]	0.647	0.01
	MTP 1	DF/PF	8.0 ± 3.0	12.6 ± 10.1	−4.6 [−5.8; −3.4]	0.525	0.02
Midstance	Ankle	DF/PF	5.2 ± 1.4	6.1 ± 2.0	−1.0 [−1.8; −0.2]	0.167	0.08
		Inv/Eve	1.2 ± 0.6	1.2 ± 0.8	0.0 [−0.7; 0.7]	0.520	0.02
		Add/Abd	1.7 ± 1.3	1.8 ± 1.0	−0.1 [−0.9; 0.7]	0.960	0.00
	Chopart	DF/PF	1.9 ± 0.7	2.1 ± 2.0	−0.2 [−1.0; 0.6]	0.227	0.06
		Inv/Eve	1.3 ± 0.5	1.1 ± 0.6	0.2 [−0.4; 0.8]	0.469	0.02
		Add/Abd	0.7 ± 0.5	1.0 ± 0.5	0.3 [−0.3; 0.9]	0.048	0.15
	Lisfranc	DF/PF	1.2 ± 0.7	1.5 ± 1.0	0.2 [−0.5; 0.9]	0.274	0.05
		Inv/Eve	0.7 ± 0.5	0.7 ± 0.4	0.0 [−0.6; 0.6]	0.841	0.00
		Add/Abd	1.1 ± 0.8	1.0 ± 0.6	0.1 [−0.6; 0.8]	0.760	0.00
	MTP 1	DF/PF	4.0 ± 3.5	5.5 ± 2.2	−1.5 [−2.5; −0.5]	0.062	0.14
Terminal stance	Ankle	DF/PF	2.6 ± 1.2	3.6 ± 1.1	−1.0 [−1.8; −0.2]	0.045	0.15
		Inv/Eve	3.6 ± 1.3	3.8 ± 1.2	−0.2 [−1.0; 0.6]	0.765	0.00
		Add/Abd	3.7 ± 1.8	4.5 ± 2.5	−0.9 [−1.8; 0.0]	0.633	0.01
	Chopart	DF/PF	5.5 ± 1.5	3.1 ± 1.3	2.3 [1.5; 3.1]	0.038	0.16
		Inv/Eve	3.2 ± 1.2	2.7 ± 1.5	0.6 [−0.2; 1.4]	0.116	0.10
		Add/Abd	1.4 ± 0.7	2.1 ± 1.0	−0.6 [−1.3; 0.1]	0.088	0.11
	Lisfranc	DF/PF	3.2 ± 1.3	3.5 ± 1.3	−0.4 [−1.2; 0.4]	0.828	0.00
		Inv/Eve	1.9 ± 0.9	2.3 ± 1.1	−0.3 [−1.0; 0.4]	0.514	0.02
		Add/Abd	2.6 ± 1.9	2.5 ± 1.1	0.2 [−0.6; 1.0]	0.137	0.09
	MTP 1	DF/PF	11.7 ± 4.8	13.3 ± 5.6	−1.7 [−2.8; −0.6]	0.198	0.07
Pre-swing	Ankle	DF/PF	12.7 ± 3.5	19.4 ± 6.1	−6.7 [−7.8; −5.6]	0.025	0.19
		Inv/Eve	2.3 ± 1.5	2.6 ± 1.5	−0.3 [−1.1; 0.5]	0.334	0.04
		Add/Abd	2.3 ± 1.5	4.8 ± 2.6	−2.5 [−3.4; −1.6]	0.011	0.23
	Chopart	DF/PF	9.5 ± 2.1	12.9 ± 4.1	−3.4 [−4.4; −2.4]	0.033	0.17
		Inv/Eve	3.4 ± 1.8	2.8 ± 1.7	0.6 [−0.3; 1.5]	0.969	0.00
		Add/Abd	3.1 ± 1.6	5.9 ± 3.2	−2.7 [−3.6; −1.8]	0.028	0.18
	Lisfranc	DF/PF	4.0 ± 1.8	3.3 ± 2.2	0.8 [−0.1; 1.7]	0.111	0.10
		Inv/Eve	2.1 ± 1.4	3.1 ± 1.9	−1.0 [−1.8; −0.2]	0.203	0.06
		Add/Abd	5.9 ± 2.5	4.2 ± 1.8	1.7 [0.8; 2.6]	0.060	0.13
	MTP 1	DF/PF	19.0 ± 6.6	23.2 ± 11.1	−4.2 [−5.5; −2.9]	0.333	0.04

*p* values represent the outcomes of the one-way ANOVA test. BMI had no significant effect except a trend towards significance for Lisfranc Inv/Eve during pre-swing (*p* = 0.035); significance: *p* < 0.0125, trend to significance. Abbreviations: MTP 1, first metatarsophalangeal joint; DF/PF, dorsal flexion–plantar flexion (sagittal plane); Inv/Eve, inversion–eversion (frontal plane); Add/Abd, adduction–abduction (transverse plane).

**Table 5:** Cross-correlation for all three cohorts.

	Contralateral Side	Affected Foot	Control Group
Ankle Inv/Eve–Ankle Add/Abd	0.623 ± 0.3	0.569 ± 0.2	0.873 ± 0.1
Ankle Inv/Eve–Forefoot DF/PF	−0.689 ± 0.3	−0.684 ± 0.2	−0.901 ± 0.1
Ankle Inv/Eve–Forefoot Inv/Eve	−0.666 ± 0.2	−0.634 ± 0.2	−0.791 ± 0.3
Ankle Inv/Eve–Forefoot Add/Abd	−0.118 ± 0.5	−0.135 ± 0.5	−0.147 ± 0.5

Abbreviations: DF/PF, dorsal flexion–plantar flexion (sagittal plane); Inv/Eve, inversion–eversion (frontal plane); Add/Abd, adduction–abduction (transverse plane).

## Data Availability

Not applicable.
